# Supplementary material for: Long-lived spin waves in a metallic antiferromagnet
Source: Nat Commun. 2023 Sep 5;14:5422. doi: 10.1038/s41467-023-40963-x (PMC10480465; doi:10.1038/s41467-023-40963-x)
Supplement: Supplementary file 1 — Supplementary Information [file 41467_2023_40963_MOESM1_ESM.pdf]

Long-lived spin waves in a metallic antiferromagnet
# – Supplementary Information –

G. Poelchen,[1,2,3,*] J. Hellwig,[4] M. Peters,[4] D. Yu. Usachov,[5] K. Kliemt,[4] C. Laubschat,[2] P. M. Echenique,[5,6] E. V. Chulkov,[5,7,8] C. Krellner,[4] S. S. P. Parkin,[9] D. V. Vyalikh,[5,6] A. Ernst,[10,9] and K. Kummer[1,†]

[1] *European Synchrotron Radiation Facility, 71 Avenue des Martyrs, 38043 Grenoble, France*
[2] *Institut für Festkörper- und Materialphysik, Technische Universität Dresden, 01062 Dresden, Germany*
[3] *Max Planck Institute for Chemical Physics of Solids, Nöthnitzer Straße 40, 01187 Dresden, Germany*
[4] *Kristall- und Materiallabor, Physikalisches Institut, Goethe-Universität Frankfurt, Max-von-Laue Strasse 1, 60438 Frankfurt am Main, Germany*
[5] *Donostia International Physics Center (DIPC), 20018 Donostia-San Sebastián, Spain*
[6] *IKERBASQUE, Basque Foundation for Science, 48011 Bilbao, Spain*
[7] *Centro de Física de Materiales (CFM-MPC), Centro Mixto CSIC-UPV/EHU, 20018 Donostia-San Sebastián, Spain*
[8] *Departamento de Polímeros y Materiales Avanzados: Física, Química y Tecnología, Facultad de Ciencias Químicas, Universidad del País Vasco UPV/EHU, 20080 Donostia-San Sebastián, Spain*
[9] *Max-Planck-Institut für Mikrostrukturphysik, Weinberg 2, 06120 Halle, Germany*
[10] *Institut für Theoretische Physik, Johannes Kepler Universität, 4040 Linz, Austria*

I. RESISTIVITY MEASUREMENT

In recent first-principle calculations, it was suggested that $CeCo_2P_2$ is a topological Dirac semimetal [1]. One important experiment to prove or disprove such a theoretical scenario are transport measurements. Here, we present resistivity measurements on single crystalline samples of both, $CeCo_2P_2$ and $LaCo_2P_2$.

The platelet shape of the single crystals [2] makes measurements with current perpendicular to the crystallographic c direction more accessible, which are presented in Fig. S1. We have taken great care to determine the absolute value of the resistivity of $CeCo_2P_2$ as precise as possible. To this end, we have used a very thin single crystal (40 µm thick) with nearly perfect quadratic shape (see inset of Fig. S1). Using a van-der-Pauw analysis [3], we could determine reliable absolute values of the resistivity. From the temperature dependence of the resistivity and a room temperature value of about 260 µΩ cm we can clearly demonstrate that $CeCo_2P_2$ is a metal. Comparing this data to the measured resistivity of $LaCo_2P_2$ we see that the room temperature resistivity of $CeCo_2P_2$ is about 3 times higher than in $LaCo_2P_2$, which might be due to the reduced density of states (DOS), as discussed in our band-structure calculations in the main part of this manuscript. Our resistivity data of $LaCo_2P_2$ agrees very well with published data in Ref. Teruya *et al.* [4], with a clear kink at the ferromagnetic ordering temperature around 135 K. For $CeCo_2P_2$, we see in addition a smooth kink in the resistivity data around 50 K. This feature is present in measurements on different samples, although its precise temperature evolution varies from sample to sample. Presently, we do not know the origin of this change in resistivity and need to analyse this further.

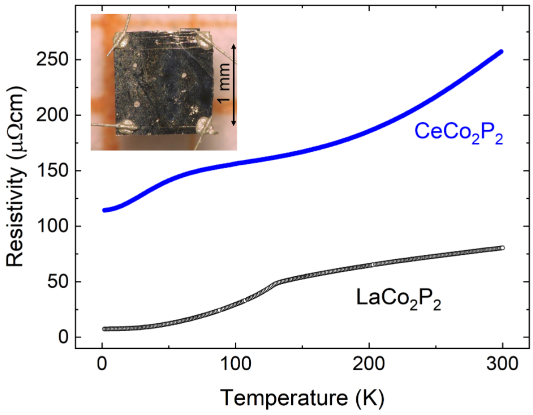

Figure S1. Resistivity as function of temperature for single crystals of $CeCo_2P_2$ (blue symbols) and $LaCo_2P_2$ (black symbols). For $CeCo_2P_2$ we used a van-der-Pauw method to extract accurate absolute values of the resistivity. The respective $CeCo_2P_2$ crystal with the four contacts made with silver paste and Pt wires is shown in the inset.

* georg.poelchen@esrf.fr
† kurt.kummer@esrf.fr

II. EFFECT OF SMALL MAGNON DAMPING ON THE RIXS SPECTRUM

As described in the main text, the measured RIXS spectra are satisfactorily described by three contributions: an elastic line at zero energy loss, a magnon excitation peak at higher energy loss, and a slowly growing, structureless background intensity coming from the tail of the fluorescence line. The elastic peak was modelled as an energy resolution limited Gaussian (FWHM $\Delta E = 28\,\mathrm{meV}$). The magnon peak was described using the usual damped harmonic oscillator model [5, 6] with damping factor γ

$$I(E_{\mathrm{loss}} > 0, \hbar\omega_q, \gamma) = I_0(\hbar\omega_q)\frac{\gamma E_{\mathrm{loss}}}{\left(E_{\mathrm{loss}}^2 - \hbar^2\omega_q^2\right)^2 + 4\gamma^2 E_{\mathrm{loss}}^2} \tag{1}$$

convolved with the experimental resolution. The energy resolution $\Delta E = 28\,\mathrm{meV}$ in our RIXS experiment was determined with high precision by carefully measuring the line width obtained from a non-resonant elastic scatterer. The results of the fit analysis using this model are shown in Fig. S2 together with the RIXS raw data acquired at different momentum transfers $\mathbf{q}$.

To discuss the effect of the resolution broadening on the possibility to experimentally determine small damping values γ, the individual contributions of the damping γ and of the resolution ΔE on the experimental magnon peak width need to be considered. For small damping ratios, i.e. $\gamma \ll \hbar\omega_q$, Eq. (1) simplifies to a Lorentzian with a FWHM of 2γ [5]. Even when γ is as small as $\Delta E/10$, the natural, lifetime limited width of the magnon excitation already causes a notable broadening of the experimentally observed peak by more than 10 %. In our case $\Delta E = 28\,\mathrm{meV}$ FWHM which allows to determine small damping factors of several meV with already good accuracy. This is well seen in Fig. S2 (bottom panel) where we compare the expected line shape of the magnon excitation for damping factors γ from 0 meV to 20 meV and the experimental data after subtraction of the elastic line and the fluorescence background.

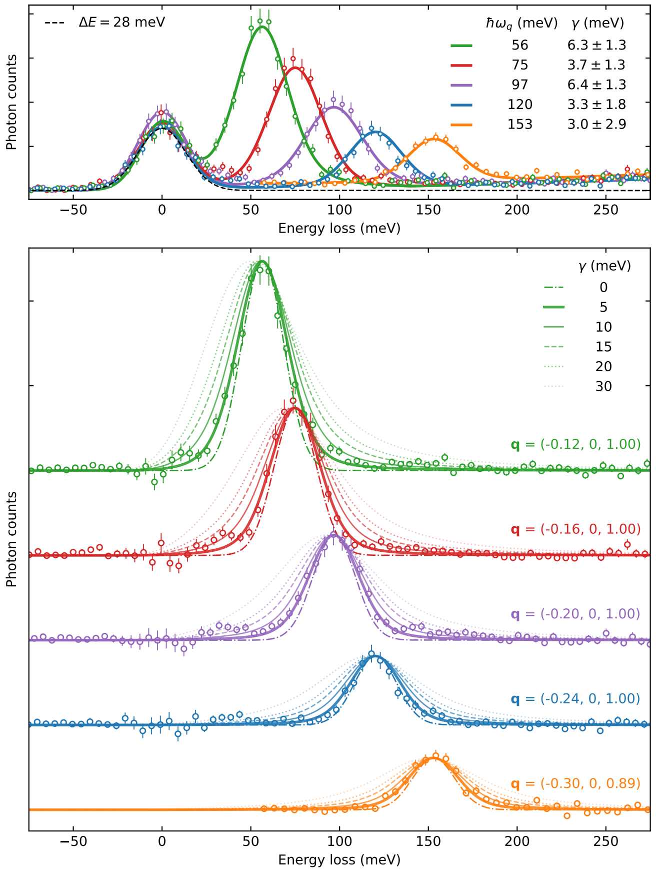

Figure S2. Top panel: RIXS raw data taken at different momentum transfers $\mathbf{q}$ together with the results of our fit analysis. Bottom panel: The same data after subtraction of a Gaussian peak with FWHM $= 28\,\mathrm{meV}$ at zero and the slowly increasing, structureless background originating from the tail of the fluorescence line peaking at about 1.5 eV energy loss. Lines show the expected line shape of the magnon excitation with increasing magnon damping γ for our energy resolution of $\Delta E = 28\,\mathrm{meV}$.

III. TEMPERATURE DEPENDENCE OF THE SPIN WAVE EXCITATIONS

In Fig. S3a, we show RIXS spectra of $CeCo_2P_2$ measured at $\mathbf{q} = (-0.24, 0, 1)$ as a function of temperature from 20 K up to 300 K. The magnon excitation is clearly visible at all measured temperatures, in line with the high magnetic ordering temperature $T_N = 440$ K. In order to quantify the evolution of the spin wave energies and intensities with temperature we fitted the data with the same model that we used to analyse the data at $T \sim 20$ K. This model does not account for thermal population of low energy excited states which causes the residual intensity in an energy window of about $\pm 2k_BT$ around the elastic line. For the damping factor γ we allowed for the same moderate linear increase with temperature that was previously reported elsewhere for THz magnons in metals [7], i.e. $\gamma(T_N) \approx 1.7 \times \gamma(T = 0)$. This increase in damping is expected due to thermal population of low energy magnons and a domain size reduction, which leads to enhanced magnon-magnon scattering and scattering at domain boundaries. In $CeCo_2P_2$ the magnon damping should increase only moderately at room temperature, by about 50 % compared to the low temperature values, due to the high Néel temperature of the material ($T_N = 440$ K). The magnon peak intensity decreases slightly with increasing temperature as well as the magnon energy at fixed $\mathbf{q}$. Spin waves commonly show a decrease in the stiffness of their dispersion with rising temperature, a behaviour that has been shown to be a result of exchange interaction renormalisation in many classes of materials [8–10].

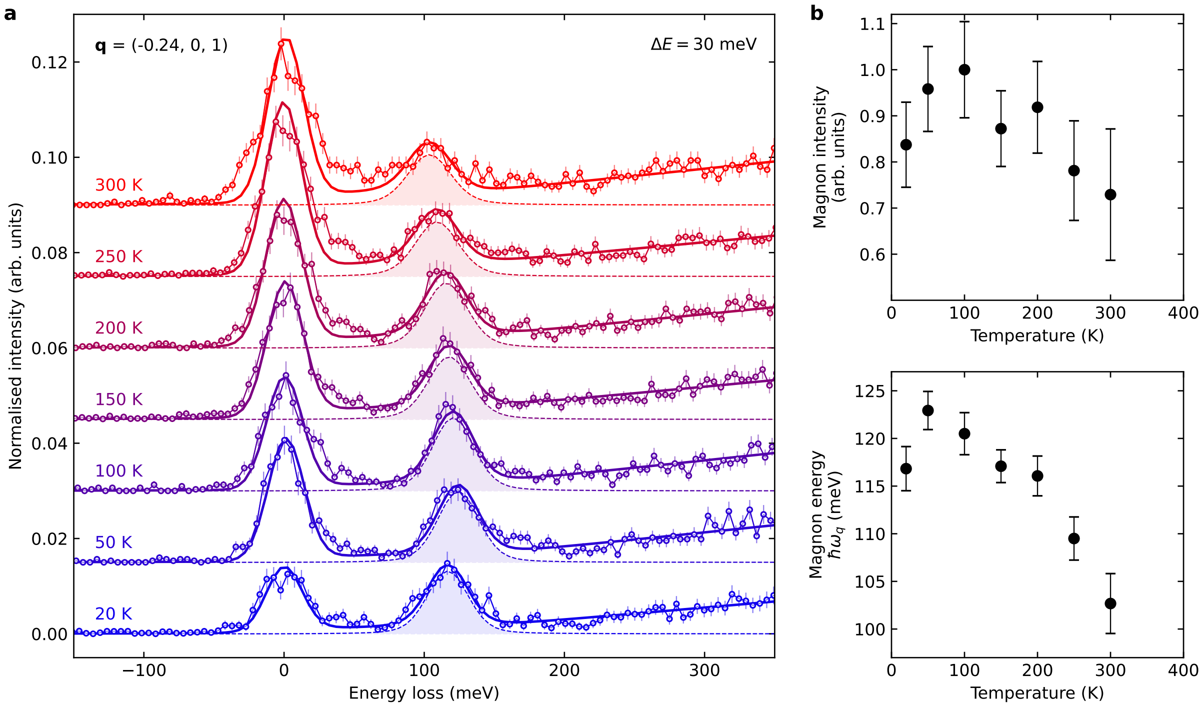

Figure S3. (a) RIXS spectra at $\mathbf{q} = (-0.24, 0, 1)$ as a function of temperature and fits to the data using the model described in Section II. The spectra are vertically offset for visualisation. The magnon peak convolved by the experimental resolution is highlighted as filled curve. (b) Temperature dependence of the intensity and excitation energy $\hbar\omega_q$ of the magnon peak.

REFERENCES

[1] Y. Xu, L. Elcoro, Z.-D. Song, B. J. Wieder, M. G. Vergniory, N. Regnault, Y. Chen, C. Felser, and B. A. Bernevig, High-throughput calculations of magnetic topological materials, Nature **586**, 702 (2020).

[2] K. Kliemt, M. Peters, F. Feldmann, A. Kraiker, D.-M. Tran, S. Rongstock, J. Hellwig, S. Witt, M. Bolte, and C. Krellner, Crystal growth of materials with the $ThCr_2Si_2$ structure type, Cryst. Res. Technol. **55**, 1900116 (2020).

[3] L. J. van der Pauw, A method of measuring specific resistivity and Hall effect of discs of arbitrary shape, Philips Res. Rep **13**, 1 (1958).

[4] A. Teruya, A. Nakamura, T. Takeuchi, F. Honda, D. Aoki, H. Harima, K. Uchima, M. Hedo, T. Nakama, and Y. Ōnuki, De Haas-van Alphen effect and Fermi surface properties in ferromagnet $LaCo_2P_2$ and related compounds, Physics Procedia **75**, 876 (2015), 20th International Conference on Magnetism, ICM 2015.

[5] Y. Y. Peng, E. W. Huang, R. Fumagalli, M. Minola, Y. Wang, X. Sun, Y. Ding, K. Kummer, X. J. Zhou, N. B. Brookes, B. Moritz, L. Braicovich, T. P. Devereaux, and G. Ghiringhelli, Dispersion, damping, and intensity of spin excitations in the monolayer $(Bi,Pb)_2(Sr,La)_2CuO_{6+\delta}$ cuprate superconductor family, Phys. Rev. B **98**, 144507 (2018).

[6] J. Zhao, D. T. Adroja, D.-X. Yao, R. Bewley, S. Li, X. F. Wang, G. Wu, X. H. Chen, J. Hu, and P. Dai, Spin waves and magnetic exchange interactions in $CaFe_2As_2$, Nat. Phys. **5**, 555 (2009).
[7] H. J. Qin, K. Zakeri, A. Ernst, and J. Kirschner, Temperature dependence of magnetic excitations: Terahertz magnons above the Curie temperature, Phys. Rev. Lett. **118**, 127203 (2017).
[8] O. W. Dietrich, J. Als-Nielsen, and L. Passell, Neutron scattering from the Heisenberg ferromagnets EuO and EuS. III. Spin dynamics of EuO, Phys. Rev. B **14**, 4923 (1976).
[9] A. Szilva, M. Costa, A. Bergman, L. Szunyogh, L. Nordström, and O. Eriksson, Interatomic exchange interactions for finite-temperature magnetism and nonequilibrium spin dynamics, Phys. Rev. Lett. **111**, 127204 (2013).
[10] D. C. M. Rodrigues, A. Szilva, A. B. Klautau, A. Bergman, O. Eriksson, and C. Etz, Finite-temperature interatomic exchange and magnon softening in Fe overlayers on Ir(001), Phys. Rev. B **94**, 014413 (2016).
